# Screen Exposure and Academic Achievement: A Systematic Review

**DOI:** 10.1002/pdi3.70071

**Published:** 2026-09-24

**Authors:** Teresa Pinheiro, Ilídia Cabral, Joana Monteiro, Virgínia Monteiro, Alberto Caldas

**Affiliations:** ^1^ School of Medicine and Biomedical Sciences Porto University Porto Portugal; ^2^ Pediatrics and Neonatology Department Entre Douro e Vouga Local Health Unit Santa Maria da Feira Portugal; ^3^ Research Centre for Human Development, Faculty of Education and Psychology Portuguese Catholic University Porto Portugal; ^4^ Colégios Ribadouro Private School Establishments Porto Portugal; ^5^ Pediatrics Department Child and Motherhood Center of the North Porto Portugal

**Keywords:** adolescent, children, pediatrics, screens

## Abstract

This systematic review examined recent evidence on how screen exposure influences the development of academic skills and educational achievement. Digital technologies have become central to children's daily lives, reshaping both learning opportunities and leisure activities. Although recreational screen use has frequently been linked to negative outcomes, the effects of educational use remain less well defined. A bibliographic search was conducted in PubMed, Scopus, Web of Science, and Mendeley for articles published between 2020 and 2024 that addressed screen use in relation to academic skills. Thirty‐one studies met the inclusion criteria. Excessive exposure was most often associated with delays in language development, poorer executive function and self‐regulation and reduced motor proficiency. In contrast, interactive and well‐structured educational applications showed potential to support literacy, numeracy, socio‐emotional development, and classroom engagement. Limitations of the available evidence include the predominance of studies in preschool populations, a lack of clear definitions of “screen exposure” and “educational tool,” and insufficient differentiation between devices and content types. The findings highlight the importance of parental and educational mediation and underscore the need for further longitudinal studies to clarify the long‐term impact of educational screen use across developmental stages.

## Graphical abstract

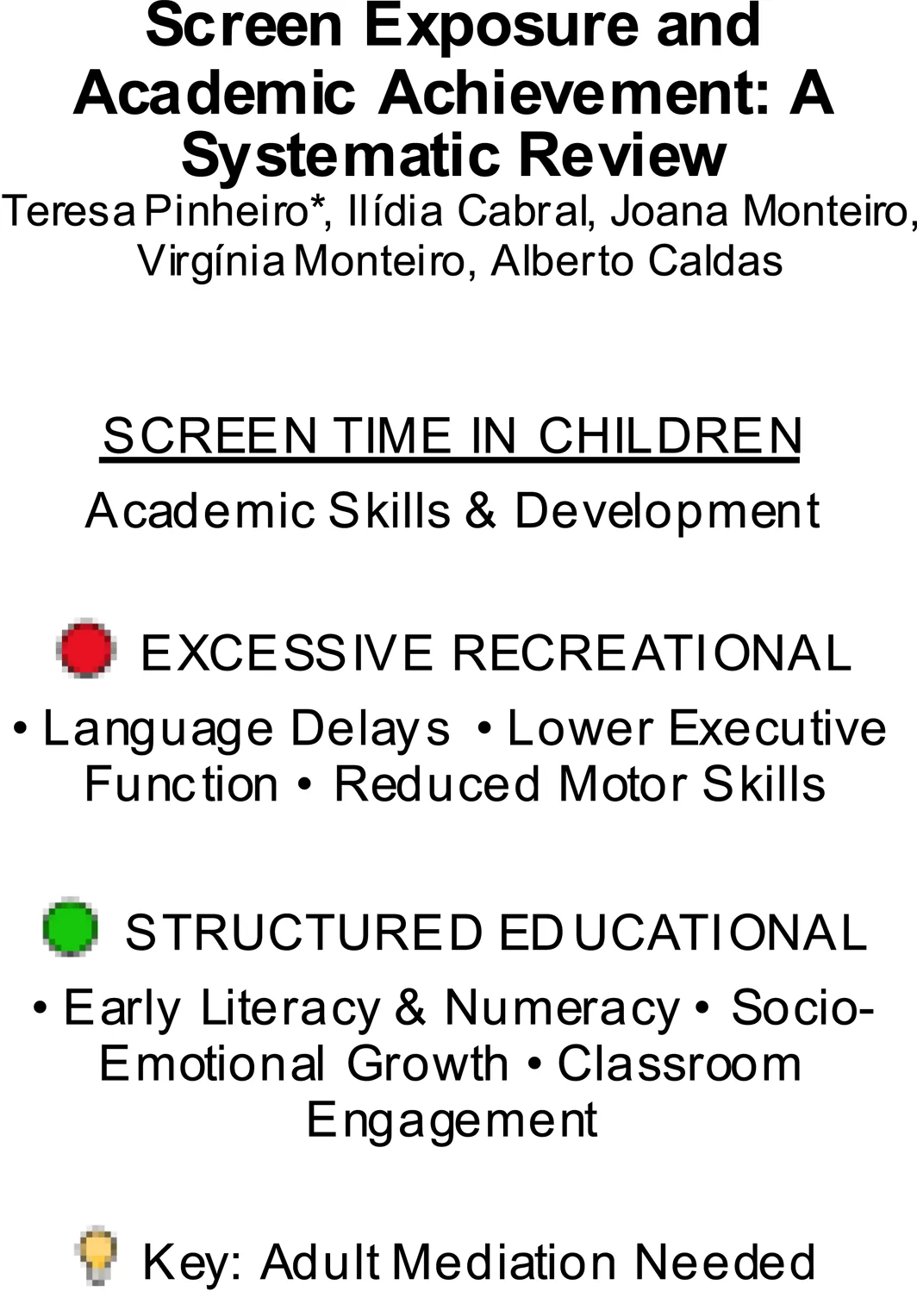

## 1 Introduction

Digital technologies have become part of children's and adolescents' daily routines, fueled by faster internet connections, the widespread use of mobile devices, and, more recently, the acceleration brought about by the COVID‐19 pandemic [1, 2, 3]. Education has not remained untouched by these shifts. In fact, the European Union responded with the *Digital Education Action Plan* (2021–2027), designed to help schools and universities navigate the digital transition while safeguarding equity and inclusiveness [1, 2, 3].

For children and adolescents, screens are now almost unavoidable. Television use has steadily declined, particularly in those under the age of eight [4, 5]. At the same time, tablets, smartphones, and laptops have taken center stage in everyday life for both young children [4] and adolescents [5, 6].

The effects of this trend are not straightforward. Screens can provide opportunities for exploration, social interaction, civic engagement, and even exposure to health‐promoting content [5]. At the same time, excessive or unsupervised use has been repeatedly linked to sleep disruption, obesity, behavioral and mood difficulties, problematic gaming or internet habits, earlier involvement in risky behaviors, cyberbullying, and poorer school outcomes [7]. From a pediatric perspective, these outcomes are closely linked to neurodevelopmental processes, executive functioning, and mental well‐being, making screen exposure a relevant topic not only for educators but also for clinicians involved in child and adolescent care.

Different clinical institutions have attempted to provide guidance. The American Academy of Pediatrics (AAP) advises avoiding screens entirely for children under 18 months (except for video calls). For children aged 2–5 years, use should be limited to about 1 hour of high‐quality content per day, preferably shared with caregivers. With older children, the emphasis shifts to context and supervision rather than strict daily limits [5]. However, these recommendations were mainly developed with recreational screen use in mind and offer limited guidance regarding screens used in educational contexts.

Since the pandemic, the educational use of screens has expanded considerably, both in and out of the classroom, and this rapid integration of digital tools into formal education has raised new questions about their impact on learning and development.

To address this issue, it is important to clarify what is meant by academic skills. The concept is broader than grades or test scores and reflects a set of competencies that allow children to engage, adapt, and succeed in school. The literature consistently highlights six interconnected domains that overlap and influence one another and may be affected differently by the type, timing, and context of screen exposure: executive functions, attention, and behavioral regulation [8, 9, 10, 11]; autonomy, self‐regulation, and adaptability [12]; literacy and numeracy [13, 14]; foundational movement skills [15, 16]; socio‐emotional development [17]; and academic achievement [13, 14, 18].

Preliminary searches in MEDLINE, JBI Evidence Synthesis, and the Cochrane Database of Systematic Reviews revealed a lack of recent systematic reviews specifically focused on the relationship between screen exposure and academic skill development in the post‐COVID context. This represents an important gap, given the profound changes in digital practices observed since 2020.

Therefore, this systematic review aims to synthesize recent evidence (2020–2024) on how different forms of screen exposure influence the development of academic skills and educational achievement in children and adolescents. In addition to summarizing existing findings, this review seeks to critically analyze inconsistencies in the literature and reflect on their relevance for both educational practice and pediatric guidance.

## 2 Review Questions

This review aims to analyze the literature concerning screen exposure and academic skills/educational achievement, and for that, the authors elaborated on some review questions (RQ):

- RQ1. What are the effects of screen exposure on children's and adolescents' academic skills?
- RQ2. How does the type of screen exposure (passive vs. active; educational vs. recreational) influence outcomes across different academic and developmental domains?
- RQ3. What is the impact of the age of onset and average daily screen time on cognitive, self‐regulatory, social, and motor development?
- RQ4. How do digital educational tools support academic development?
- RQ5. What are the main moderating factors in the relationship between screen time and academic performance?

## 3 Methods

### 3.1 Study Design

This review followed a structured approach in line with PRISMA guidelines.

### 3.2 Search Strategy

A three‐step search process was conducted to identify all relevant published studies. First, a preliminary exploration was conducted across PubMed, Scopus, Web of Science, Mendeley, Google Scholar, and SciELO to map the existing literature. Search terms combined controlled vocabulary (MeSH) with free‐text keywords, including “screen time,” “academic skills,” “academic achievement,” “children,” and “digital media.” Boolean operators were applied and tailored to each database's requirements. Titles, abstracts, and indexing terms of retrieved articles were then reviewed to refine the final strategy. In addition, reference lists of included studies were screened to identify further eligible publications.

The search period (2020–2024) was defined to reflect the substantial increase in research on children's screen use following the COVID‐19 pandemic. However, only a few of the included studies explicitly examined screen use changes in the context of the pandemic. The remaining studies were published during this period but investigated screen exposure and developmental outcomes without directly addressing pandemic‐related factors. The term “post‐COVID digital landscape” is therefore used to characterize the broader temporal and contextual backdrop of the evidence base, rather than to imply that all included studies focused on pandemic‐related changes.

### 3.3 Eligibility Criteria

Studies were eligible if published between 2020 and 2024, addressed the impact of screen exposure on the development of academic skills or educational achievement, included children or adolescents (0–18 years), and were available in full text. Eligible designs included analytical observational studies (prospective and retrospective cohorts, case–control studies, and analytical cross‐sectional studies) as well as descriptive studies (case series, case reports, and descriptive cross‐sectional studies). Systematic reviews that met the inclusion criteria were also considered, given the limited number of recent publications on this topic.

### 3.4 Study Selection

All records were imported into EndNote 21 and uploaded to Rayyan for screening. Duplicate entries were removed automatically. Titles and abstracts were reviewed by the authors against predefined criteria, and the full texts of potentially relevant studies were retrieved for independent assessment.

### 3.5 Data Extraction

Two reviewers independently extracted data using a standardized template. Information collected included authorship and publication year, study setting, design, participant characteristics, type and context of screen exposure (educational vs. recreational; active vs. passive), duration of use, outcomes assessed (e.g., language, literacy, executive function, attention, motor skills, or academic achievement), potential moderators, and main results.

### 3.6 Risk of Bias and Certainty Assessment

The methodological quality of the included studies was assessed using validated tools appropriate to each study design. Observational studies (cross‐sectional, prospective cohort, and retrospective cohort designs) were evaluated using the Newcastle–Ottawa Scale (NOS) and systematic reviews included were appraised using the AMSTAR‐2 instrument. Formal assessment of the certainty of evidence was not undertaken, as this review did not generate clinical recommendations. Assessment of publication bias was not feasible given the absence of a quantitative meta‐analysis and the considerable heterogeneity across included studies.

### 3.7 Data Synthesis

Because of the methodological heterogeneity of the included studies, a meta‐analysis was not feasible and the results were synthesized thematically.

## 4 Results

**FIGURE 1:**
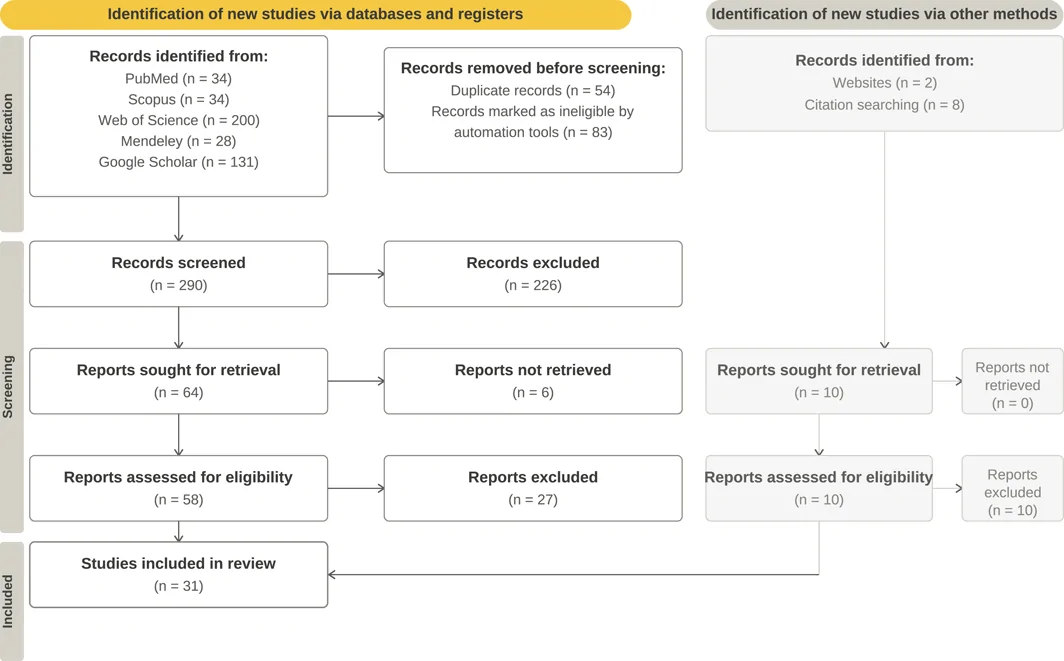
PRISMA flow diagram.

In total, 290 records were identified through database searches and reference screening. After the removal of duplicates and application of the eligibility criteria, 31 studies were included in the final analysis (Figure 1). The evidence was characterized by considerable heterogeneity across age groups, exposure definitions, tools, and outcome measures, which precluded a quantitative meta‐analysis and justified a thematic synthesis approach.

### 4.1 Study Characteristics

A total of 31 studies published between 2020 and 2024 were included in this review. Among the primary studies, sample sizes ranged from 52 to 10.370 participants, reflecting a diverse range of evidence. Geographically, the included literature spans multiple continents, with studies conducted in China, Poland, Turkiye, Russia, Pakistan, Israel, Italy, the United States, and Canada.

Observational studies constitute the core of the review, predominantly employing cross‐sectional designs and longitudinal cohort studies. Regarding secondary evidence, the review incorporates systematic reviews, some of which include quantitative meta‐analyses that consolidate effect sizes for specific domains. The framework is further enriched by narrative and bibliographic reviews and a Master's thesis, which provide essential pedagogical and exploratory context.

The synthesized evidence predominantly focused on early childhood development, with a significant concentration of research targeting the preschool population (infants, toddlers, and children under 6 years of age). A smaller portion of the evidence base addresses school‐aged children and adolescents, whereas mixed‐age samples are primarily found in broad systematic reviews.

The vast majority of the studies evaluated mixed screen exposure, which encompasses total daily duration across multiple platforms, including television, smartphones, and tablets. Conversely, only a marginal fraction of the literature isolated specific devices, such as mobile‐only usage.

The impact of screen exposure is multidimensional, spanning several critical developmental domains. Executive function and self‐regulation emerge as the most frequently investigated outcomes, followed closely by language and literacy development. Additionally, the evidence base explored academic achievement and social‐emotional development, whereas a growing subset of studies examined motor skills and the risks associated with problematic media use.

Risk of bias was assessed for all included studies using validated instruments appropriate to each study design. Most studies appraised with the NOS scale were rated at low risk of bias (score 7–9), and only a small number of studies were rated as moderate risk (score 5–6). No study was rated as high risk. The most frequent sources of bias were reliance on parental‐reported screen time, the absence of objective exposure measures, and cross‐sectional designs that preclude causal inference. Systematic reviews were appraised using AMSTAR‐2. Three were rated as high confidence, three as low confidence, and one as critically low confidence, primarily due to the absence of a pre‐registered protocol, inadequate risk of bias assessment of included studies, and failure to account for publication bias in the interpretation of results. Two additional studies, a master's thesis [42] and a narrative bibliographic review [39], were not formally appraised, given their nonpeer‐reviewed or non‐systematic nature.

### 4.2 Executive Functions, Attention, and Behavioral Skills

Executive functions, attention, and behavioral skills are fundamental to a child's capacity to engage in learning, regulating emotions, and maintain task‐focused behavior in the classroom [19]. This domain was the most frequently assessed, reflecting its critical role in neurocognitive development and school readiness.

The synthesis of the evidence indicates a predominantly negative association between screen exposure and executive functioning, particularly regarding inhibitory control and self‐regulation in preschool‐aged children [19, 20, 21, 22, 23, 24, 25, 41]. Longitudinal data suggested a “sensitive period” effect in which earlier exposure is more strongly predictive of later deficits than concurrent use [23].

However, the impact appeared to be modulated by interactivity and content quality rather than duration alone [40]. Although passive consumption and adult‐directed content were consistently linked to poorer attention regulation [25, 27, 30], interactive educational tools used in structured settings were associated with positive classroom behaviors [28, 31]. Conversely, unsupervised mobile device use was more strongly associated with self‐regulation deficits than traditional television, potentially due to the displacement of sensorimotor play. Parental mediation emerged as a significant protective factor. The data distinguish between background or unsupervised exposure, which correlated with behavioral dysregulation, and co‐viewing or guided use, which mitigated these risks [25, 26, 27]. Methodologically, the minority of studies reporting null associations suggested that screen time often acts as a proxy for other health determinants, as excessive use may indirectly impair executive function by disrupting sleep patterns and reducing physical activity [32, 33, 43].

A minority of investigations reported no significant associations between screen time and later emotional dysregulation or behavioral problems [20]. These inconsistencies may reflect methodological differences, particularly reliance on parental reports, variations in exposure definitions, and limited control for contextual factors such as parental mediation, home environment, and socioeconomic background.

### 4.3 Autonomy

Autonomy, defined as the ability to initiate, regulate, and sustain learning‐related behaviors independently, is an essential component of self‐directed learning and academic resilience. It was the least explored domain in the literature we reviewed.

Most available evidence came from studies focusing on digital educational tools, particularly artificial intelligence (AI)‐based or adaptive learning platforms [12], but remained fragmented due to varying conceptualizations.

Importantly, technology use does not automatically promote autonomy, as poorly structured digital environments may, conversely, foster passive learning or dependence on automated feedback systems [12]. Therefore, the impact of digital tools on autonomy appears to be highly dependent on content quality, pedagogical design, and the extent of adult mediation.

### 4.4 Literacy and Numeracy

Literacy and numeracy are fundamental academic skills that provide the basis for most learning activities in formal education and are closely linked with early cognitive and language development.

The findings are inconsistent [12, 23, 34, 35], highlighting the critical influence of content type and interactivity over duration alone. Although excessive daily exposure (exceeding 2–3 hours) correlates with language delays in preschoolers [12], passive viewing and fast‐paced entertainment appear particularly detrimental to linguistic outcomes [24, 27]. In contrast, interactive educational applications and adaptive AI tools, especially when guided by adult mediation, demonstrate potential to support literacy skills and reduce performance anxiety [12, 28, 29]. The evidence also suggests specific vulnerabilities, such as reduced phonological memory in boys exposed to passive gaming [30], and indicates a bidirectional relationship among older students in which lower prior achievement may drive increased screen engagement [36].

### 4.5 Foundational Movement Skills and Fine and Gross Motor Skills

Foundational movement skills (FMS) are essential for children's physical development and school readiness, as they support fine motor skills, such as writing, and gross motor skills related to play and physical engagement [15, 24].

The impact of screen exposure on FMS is characterized by a stark contrast between passive consumption and active engagement. Excessive and predominantly passive exposure was consistently associated with poorer motor development, particularly in younger children [15, 24, 32]. This negative association is largely attributed to the displacement hypothesis, where sedentary behaviors and repetitive touchscreen gestures replace complex sensorimotor play [32]. Conversely, digital tools specifically designed to promote physical interaction, such as exergames and motor‐oriented educational apps, demonstrated positive effects on balance and coordination when integrated into structured learning contexts [15].

### 4.6 Socio‐Emotional Development

Socio‐emotional development includes children's ability to comprehend and manage emotions, foster relationships, and demonstrate empathy and resilience. These skills are vital for collaborative learning, classroom conduct, and overall psychological health.

The impact of screen exposure on socio‐emotional development is highly context‐dependent, driven more by the nature of the interaction than by duration. High levels of passive or unstructured exposure, particularly before age two, are consistently associated with deficits in emotional regulation and lower engagement in social play [19, 23, 25, 27, 31]. These negative outcomes appear largely mediated by the displacement of parent–child interactions and real‐world peer opportunities. However, parental mediation serves as a critical protective factor as co‐viewing and shared activities can mitigate neurodevelopmental risks associated with screen use [20, 25, 27, 28].

Furthermore, when used intentionally, such as through collaborative educational apps or for maintaining social connections in adolescence, digital tools can support empathy and reduce loneliness, provided the usage is structured and distinct from passive consumption [28].

### 4.7 Academic Achievement

Academic achievement represents a global outcome of multiple interacting factors, including cognitive abilities, emotional regulation, behavior, and environmental influences. It reflects the cumulative impact of a child's development across various domains on school performance and is typically expressed through grades or standardized assessments.

The relationship between screen exposure and academic achievement is characterized by a distinct dichotomy between recreational and educational use. Although excessive recreational exposure is consistently associated with lower grades and reduced school engagement, purpose‐driven educational use demonstrates potential positive outcomes [29, 36, 37]. The concept of problematic media use (PMU) emerged as a critical mediator, where the pattern of consumption, linked to sleep disturbances and behavioral difficulties, predicted underachievement more accurately than duration alone [37, 38]. Although daily exposure exceeding two to 3 hours represents a risk threshold, the evidence suggests a bidirectional relationship, where students with lower prior achievement are more prone to increasing screen engagement over time [36, 37]. Additionally, gender‐specific patterns were observed, with academic risks associated primarily with video gaming in boys and social media use in girls [36].

## 5 Discussion

The impact of screen‐based technologies on children's development is neither uniform nor easily reducable to simple cause–and–effect relationships. Prolonged or passive screen time is frequently associated with difficulties in language development, executive functioning, self‐regulation, and motor skills. Interactive, well‐designed tools can foster learning, social interaction, and cognitive growth. However, outcomes vary depending on device type, content, duration, level of engagement, and the extent to which screen use is balanced with activities that support health and well‐being.

A key problem in the existing literature is the conceptual and methodological heterogeneity. Definitions of “screen exposure” differ widely and many studies rely on retrospective parental reports, and outcome measures are far from standardized.

Nevertheless, three theoretical mechanisms seem to underlie many of the negative associations reported: the displacement hypothesis, where screen time replaces activities essential for development (e.g., free play, reading, and physical activity); the cognitive overstimulation hypothesis, suggesting that fast‐paced or highly stimulating content may overload immature attentional systems; and the reduced social interaction hypothesis, particularly relevant in early childhood, where excessive screen exposure can limit parent–child and peer interactions crucial for language and socio‐emotional development. Taken together, these mechanisms highlight that the impact of screen exposure depends less on duration per se and more on what screens replace, how they are used, and in what developmental period.

### 5.1 Educational Versus Recreational Exposure

One of the clearest patterns emerging from this review is the distinction between recreational and educational screen exposure. Recreational screen use, especially when passive, prolonged, and initiated at an early age, was more consistently associated with adverse outcomes across multiple domains. In contrast, structured and purposeful educational screen use showed potential benefits, particularly for literacy, numeracy, and collaborative skills, when aligned with curricular goals and accompanied by adult guidance. However, these positive effects were highly contingent on content quality, pedagogical design, and context of implementation.

Importantly, digital tools should not be viewed as inherently beneficial or harmful. Their impact depends on how they are embedded within broader learning and developmental environments. This reinforces the need for critical selection of digital resources in both school and home settings.

### 5.2 Relevance for Pediatric Practice

From a pediatric perspective, the findings of this review have direct implications for clinical practice.

Screen exposure is not merely an educational concern, but a developmental and health concern, closely linked to neurocognitive development, emotion regulation, sleep quality, and mental well‐being. Children presenting with attention difficulties, behavioral dysregulation, sleep problems, or academic underachievement should be routinely assessed for their patterns of screen use, including type of content, timing of exposure, and context. Importantly, pediatric guidance should move beyond simple time‐based recommendations and emphasize qualitative aspects of screen exposure (encouraging age‐appropriate content, promoting co‐viewing and parental mediation, ensuring a balance with physical activity and sleep, and actively discouraging early, unstructured, and excessive exposure).

### 5.3 Gaps and Future Research Directions

This review identified important gaps in the current literature: most studies focus on preschool children, with a remarkable underrepresentation of adolescents, despite this group having the highest levels of digital exposure; autonomy and self‐regulated learning remain insufficiently explored; and most studies rely on subjective measures of screen use. Future research should incorporate objective digital exposure tracking and longitudinal designs.

Expanding the temporal scope to include pre‐pandemic data may enable more robust comparisons of digital practices before and after COVID‐19. This would help clarify which observed effects reflect structural changes in exposure versus contextual disruptions caused by the pandemic.

Future research should aim not only to document associations but to clarify causal pathways and identify protective factors that mitigate potential risks.

## 6 Conclusion

This systematic review highlights that screen exposure has a multidimensional and context‐dependent impact on children's academic development. Although early, excessive, and predominantly passive screen use is more consistently associated with poorer executive functioning, language outcomes, self‐regulation, and academic achievement, not all screen exposure is detrimental. Structured, purposeful, and age‐appropriate educational use, particularly when guided by adults, may support learning processes in selected domains.

However, the current evidence base remains limited by methodological heterogeneity, an overrepresentation of preschool populations, the lack of standardized exposure definitions, and the scarcity of objective screen use measurements. Autonomy and self‐regulated learning, despite their relevance in modern educational contexts, remain underexplored.

From a pediatric standpoint, screen exposure should be approached as a developmental and health‐related factor rather than solely as an educational variable. Clinical guidance must move beyond quantitative time limits and focus on qualitative dimensions of use, including content type, context, timing, and co‐use with caregivers.

Future research should prioritize longitudinal designs, include older age groups, integrate objective exposure measures, and evaluate not only risks but also protective factors and optimal models for integrating digital tools into children's learning environments. Until then, a balanced and individualized approach remains essential to support children's academic, cognitive, and socio‐emotional development in an increasingly digital world.

## Author Contributions

**Teresa Pinheiro:** conceptualization, methodology, data curation, formal analysis, writing – original draft, and project administration. **Ilídia Cabral:** methodology, validation, data curation, writing – review and editing. **Joana Monteiro:** literature search, data extraction, writing – review and editing. **Virgínia Monteiro:** literature search, data curation, writing – review and editing. **Alberto Caldas:** supervision, critical review, writing – review and editing.

## Funding

The authors have nothing to report.

## Ethics Statement

The authors have nothing to report.

## Consent

The authors have nothing to report.

## Conflicts of Interest

The authors declare no conflicts of interest.

## Data Availability

The data that support the findings of this study are available from the corresponding author upon reasonable request.
